# Infection of Cultured Human Endothelial Cells by *Legionella pneumophila*

**DOI:** 10.1371/journal.pone.0002012

**Published:** 2008-04-23

**Authors:** Lucius Chiaraviglio, Daniel A. Brown, James E. Kirby

**Affiliations:** Department of Pathology, Division of Cancer Biology and Angiogenesis, Beth Israel Deaconess Medical Center, Boston, Massachusetts, United States of America; Columbia University, United States of America

## Abstract

*Legionella pneumophila* is a gram-negative pathogen that causes a severe pneumonia known as Legionnaires' disease. Here, we demonstrate for the first time that *L. pneumophila* infects and grows within cultured human endothelial cells. Endothelial infection may contribute to lung damage observed during Legionnaires' disease and to systemic spread of this organism.

## Introduction

*Legionella pneumophila* causes an estimated 2–15% of community acquired pneumonia requiring hospitalization [1]. *L. pneumophila* is a gram-negative pathogen whose primary ecological niche appears to be as a parasite of protozoa. However, humans and other animals may become secondarily infected after inhaling or aspirating organisms.

Fascinatingly, the cell biology of infection of protozoa and humans cells appears remarkably similar. On entering both macrophages [2] and protozoa [3], [4], *L. pneumophila* inhibits phagolysosome fusion [5] and multiplies in a compartment having properties of endoplasmic reticulum [6]. Escape from phagolysosome fusion, intracellular multiplication, and lysis of the host cell relies on a type IV secretion system encoded by the *dot/icm* gene complex [7]–[9].

Resident alveolar macrophages appear to be a Trojan horse for human infection [10]. *L. pneumophila* infects these cells and continues to grow within macrophages recruited during the subsequent inflammatory response. Infection may then spread to alveolar epithelial cells that line the airways, supported by observations of growth in primary [11] and transformed type I [12] and II pneumocytes [13], and growth of the related *L. dumoffii* in alveolar epithelial cells *in vivo* [14].

Bacterial dissemination does not stop at the alveolar wall. In both human autopsy studies [15] and animal models [16], *L. pneumophila* spreads to multiple organs. This suggests that the organism may infect and breach the remaining anatomic barrier in the lung — pulmonary blood vessels — as a potential initiating event in systemic spread of the organism via the blood stream. Here, we begin to address this hypothesis through *in vitro* study of the interaction of *Legionella pneumophila* with primary human endothelial cells.

## Results

### Infection of endothelial cells

**Figure 1:**
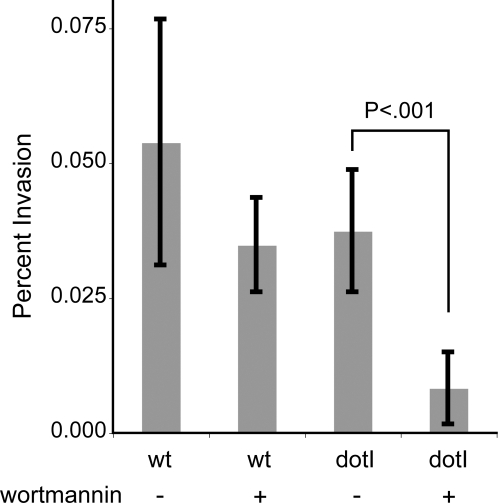
*Legionella pneumophila* invades into endothelial cells. *L. pneumophila* strains Lp01 (wt) and an isogenic *dotI* mutant were seeded onto HUVEC at a ratio of approximately 300∶1. After a two hour incubation in the presence or absence of 100 nM wortmannin, gentamicin treatment was used to kill extracellular bacteria, and then intracellular bacteria were enumerated. Intracellular bacteria are expressed as percentage of the original titered inoculum to determine the invasion efficiency. Results shown are the mean and standard deviation of sextuplicate assays and are representative of two independent experiments.

We first tested whether *Legionella pneumophila* strain Lp01 [17] invades into primary human umbilical vein endothelial cells (HUVEC). Using a gentamicin protection assay, we assessed whether bacteria were protected from gentamicin [18], a polar antibiotic that penetrates slowly into eukaryotic cells and does not kill internalized bacteria during the time frame of the assay. Using this procedure, we found that *L. pneumophila*, strain Lp01, reproducibly invades into endothelial cells (see Fig. 1) with approximately 0.05% of bacteria internalized during a 2 hour incubation. The ability of *L. pneumophila* to invade into endothelial cells was confirmed by fluorescent microscopy where internalized bacteria were distinguished by differential staining as described in detail below.

Of note, the invasion efficiency was roughly 100 fold lower than what was previously observed for primary macrophages [19], an expected result since endothelial cells are not professional phagocytes. We compared invasion efficiency to the *L. pneumophila*, isogenic *dotI* mutant, HL051C [20], which was previously shown to be defective in inhibition of phagolysosome fusion and intracellular replication in macrophages. Although a trend towards slightly lower invasion efficiency by HL051C was noted, this did not reach statistical significance (Fig. 1). Previously, invasion of a *dot/icm* mutant, but not wild type *L. pneumophila* was shown to be blocked by the PI-3 kinase inhibitor, wortmannin [21], implying that invasion of wild type and mutant bacteria occur through distinct pathways. We found the same to be true in endothelial cells (Fig. 1). Here, treatment with 100 nM wortmannin led to a 4.5 fold reduction in invasion of HL051C (*P* = 0.005) vs. an insignificant reduction of 1.5 fold for Lp01. Therefore, since wortmannin did not substantially block invasion of wild type bacteria, invasion of wild type and the *dotI* mutant appears to occur by distinct pathways as described previously for macrophages [21], [22].

### Replication of bacteria within endothelial cells

**Figure 2:**
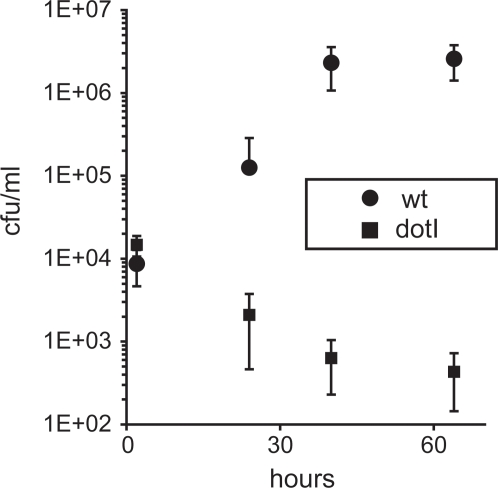
*L. pneumophila* grows inside of primary human endothelial cells. Growth curves of *L. pneumophila* Lp01 (wt) and an isogenic *dotI* mutant after infection of HUVEC. At indicated time points after infection, the numbers of intracellular and extracellular bacterial colony forming units (cfu) were determined and added together. As bacteria do not multiply extracellularly, the total number of bacteria reflects intracellular growth. Data points are the mean and standard deviation of three separate assays.

We next tested whether *L. pneumophila* replicates inside of endothelial cells. As shown in Fig. 2, we found that wild type Lp01 replicates to high levels in HUVEC with a two to three log growth increase, leading to lysis of host cells and release of bacteria (data not shown). In contrast, the *dotI* mutant, HL051C, failed to grow and was gradually eliminated. These results are consistent with previous observations in macrophages and suggest that growth is dependent on the *dot/icm*-dependent, type IV secretion system [17], [23]. These observations were confirmed by fluorescent microscopy where large numbers of Lp01 were found intracellularly at later time points vs. the infrequent appearance of HL051C. The latter often appeared to have degraded nucleoids by DAPI staining.

Similar growth curves were observed for other endothelial cell types tested including primary human lung microvascular and umbilical artery endothelial cells (data not shown). The ability to grow in the diverse types of endothelial cells surveyed is especially interesting as they are known to have diverse physiological properties [24]. Bacteria also grew within primary pulmonary artery endothelial cells of bovine origin (a species that also develops Legionnaires' disease [25]), suggesting that the ability to infect endothelial cells crosses species boundaries.

### Altered phagosome properties

**Figure 3:**
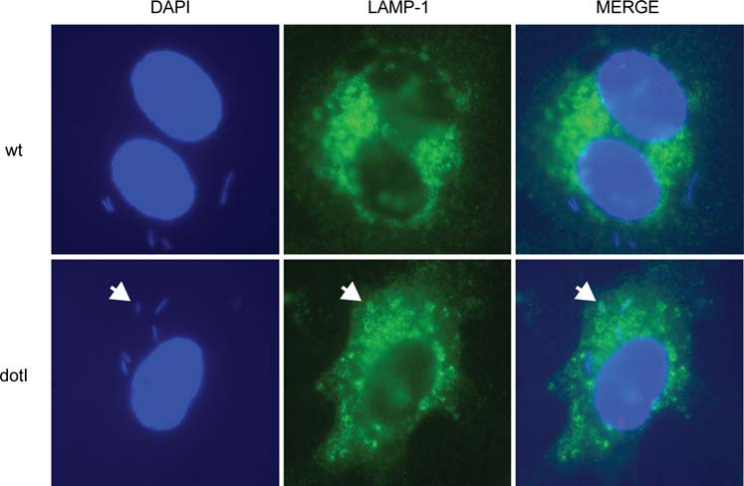
*L. pneumophila* inhibits endothelial phagolysosome fusion. Representative images showing lysosomal targeting of bacteria 1.5 hours after infection of HUVEC at a bacterial to endothelial cell ratio of approximately 300∶1. The *dotI* mutant strain, but not Lp01 (wt), show extensive colocalization with LAMP-1, a lysosomal marker (bacteria stained blue with DAPI, LAMP-1 stained green). An example of a colocalizing bacteria is indicated with an arrow. Extracellular bacteria (not present in these images) were distinguished by immuno-labeling with a red fluorophore prior to permeabilization of endothelial cells.

We next examined whether *L. pneumophila* inhibits fusion of bacterial phagosomes with lysosomes, as observed previously during infection of macrophages[5]. Using an immunofluorescence staining technique, we assessed the colocalization of internalized bacteria with a lysosomal marker, LAMP-1. At 1.5 hours after infection, only 6% of Lp01 colocalized with LAMP-1 vs. 57% of the *dotI* mutant (*P*<0.001, Fig. 3). Therefore, only the replication-competent Lp01, but not the replication-defective *dotI* mutant, efficiently escaped from lysosomal fusion.

**Figure 4:**
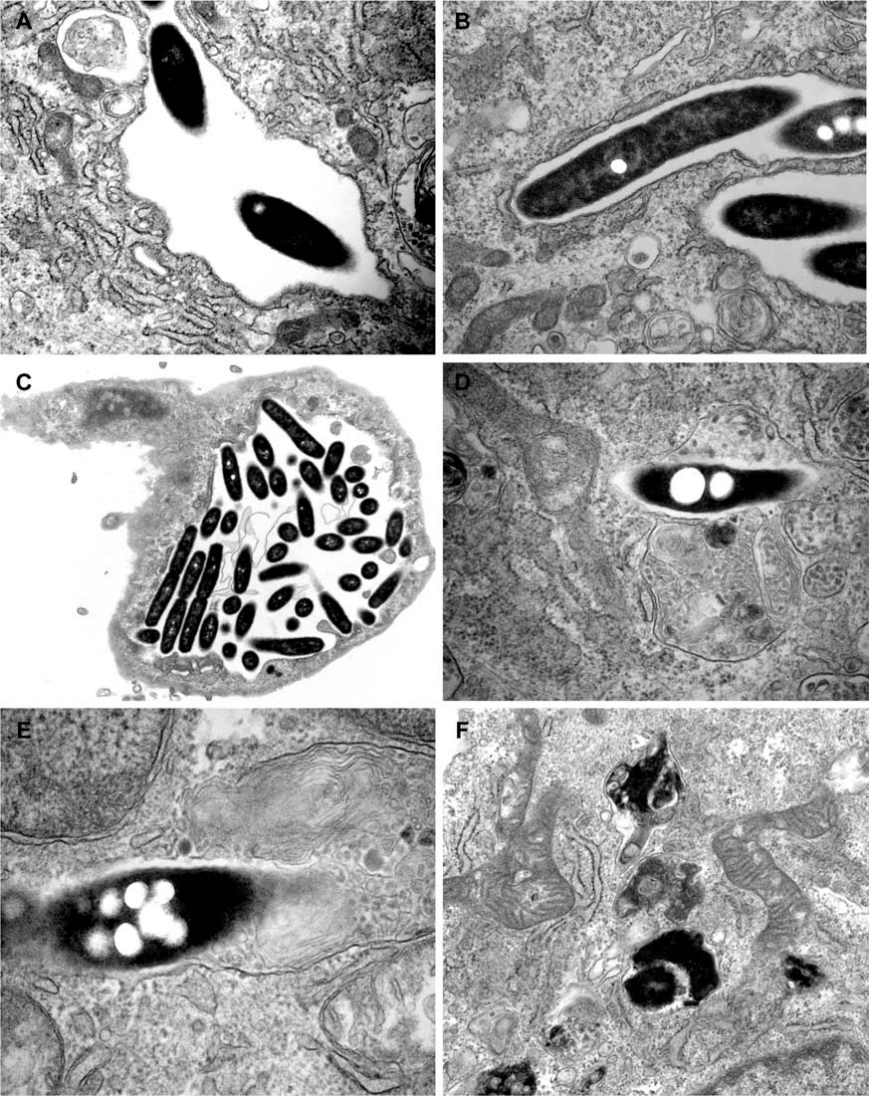
*L. pneumophila* replicates in membrane bound compartment associated with endoplasmic reticulum. (A, B) The Lp01strain is surrounded by ribosome studded endoplasmic reticulum (RER) at 6 hours after infection. (C) At 24 hours, many phagosomes are filled with large numbers of bacteria. In this image, RER association is still evident (left side of vacuole), however, at this late stage in infection, vacuolar membrane appears partially disrupted. In contrast, *dotI* mutants do not associate with RER and are surrounded by a simple, smooth membrane, at times also containing membranous debris, e.g., (D) 6 hours and (E) 24 hours. Intact bacteria were far less numerous, while electron dense structures suggestive of partially degraded bacteria were often present, (F) 6 hours. Magnifications (in figure): A, ×54,000; B, ×54,000; C ×19,000; D ×82,000; E ×119,000; F ×41,000.

We next examined the morphogenesis of *L. pneumophila* containing vacuoles by transmission electron microscopy. Within 6 hours of infection by Lp01, most bacterial phagosomes were surrounded by ribosome studded endoplasmic reticulum (RER) (Fig. 4A,B), as observed previously in infected macrophages [26] and amoeba [27]. At 24 hours after infection, organisms had multiplied in vacuoles to largely fill the endothelial cell (Fig. 4C). In contrast, the *dotI* mutant was always contained in smooth vacuoles, usually as single bacteria, and without associated RER or ribosomes at all time points (Fig. 4D, E). Furthermore, mutant bacteria often appeared degraded and were occasionally found in vacuoles containing membranous debris (Fig. 4F). Both mutant and wild type organisms were found in membrane-bound phagosomes, never freely in the cytoplasm, although disruption of the vacuolar membrane was noted late in the infection cycle of wild type organisms. Taken together, intracellular parasitism of endothelial cells appears similar to observations in macrophages.

## Discussion

Here, we for the first time identify the ability of *Legionella pneumophila* to invade and replicate inside diverse types of primary human endothelial cells, including endothelial cells obtained from the lung. We believe that this property may have special relevance for the pathogenesis of Legionnaires' disease. Previous studies have focused on the organism's ability to grow within macrophages and alveolar epithelial cells. However, the capillary network, formed by endothelial cells, also makes up a critical and potentially vulnerable part of the lung.

The biology of endothelial infection is consistent with what has been described for infection of macrophages, alveolar epithelial cells, and protozoa. The organism inhibits phagolysosome fusion and grows intracellularly in a process dependent on the *dot/icm* type IV secretion machinery. However, contact-dependent cytotoxicity observed during infection of macrophages and red blood cells [19], [28] does not occur (i.e., as determined by LDH release assay[19] after pelleting bacteria onto an endothelial monolayer for 5 minutes at 800 *g*, data not shown). This may reflect a greater resistance to osmotic lysis, differences in the interaction with the bacterial type IV secretion machinery, or decreased invasion efficiency. Nevertheless, endothelial cell death still occurs after intracellular multiplication of bacteria. It is possible that endothelial cell infection and death *in vivo* leads to damage of pulmonary capillaries and allows bacteria access to the systemic blood circulation, although lymphatic drainage of organisms might also contribute to systemic spread.

Endothelial damage may also in part account for pathologies such as hemorrhage that have been observed during Legionnaires' disease [29], [30] or adverse sequelae such as pulmonary fibrosis [31]. Here, the lung forms scar tissue rather than re-establishing surfaces capable of air exchange, leading to permanent disability after bacteriological cure [31]. Interestingly, patients with prosthetic heart valves appear to be at particular risk of developing endocarditis from *Legionella* [32]–[34]. Possibly, this relates to a susceptibility of endothelial cells overlying prosthetic material to infection with this organism.

Clearly, the extent and consequences of endothelial infection by *L. pneumophila* need to be examined using *in vivo* models. However, the vascular endothelium should now be considered a potential participant in the pathogenesis of Legionnaires' disease.

## Materials and Methods

### Bacterial strains and infections

*Legionella pneumophila* strains were described previously [17]. Endothelial cells were purchased from Cambrex and passaged in EGM2-MV medium from the same manufacturer. To perform infections, bacteria were grow in AYE medium to early stationary phase, resuspended in EGM-2MV, and used to infect endothelial cells grown on collagen I (Vitrogen, Cohesion, Inc.) treated twenty-four well dishes.

### Assessment of bacterial invasion

After infection at a bacterial to endothelial cell ratio of approximately 300∶1, extracellular bacteria were killed by a 45 minute treatment with 100 µg/ml of gentamicin, and surviving intracellular bacteria enumerated after eukaryotic cell lysis with 0.1% saponin. Statistical comparisons were performed using JMP IN Vs. 5.1.2 (SAS Institute, Cary, NC) by the Wilcoxon Ranked Sum Test for continuous and Fisher's Exact Test for categorical variables with *P*<0.05 considered significant.

### Assessment of bacterial replication

At multiple time points after infection, both extracellular and intracellular bacteria were enumerated by sampling extracellular medium and then intracellular compartments after saponin lysis. For bacterial replication experiments only, the infectious inoculum was harvested from αBCYE plates rather than from early stationary phase liquid cultures used for all other assays.

### Endocytic trafficking assay

To assess fusion of *L. pneumophila* containing phagosomes with lysosomes, endothelial cells were plated on collagen I-treated, German glass coverslips (Bellco Glass) and infected at a ratio of approximately 300 bacteria per endothelial cell. Extracellular bacteria were stained prior to permeabilization with a rabbit anti-*Legionella* primary antibody (gift of Ralph Isberg, Tufts Medical School) followed by a Alexa-594-labeled goat anti-rabbit secondary antibody. After permeabilization with 0.2% Triton®-X 100 for 15 minutes, lysosomal compartments were stained with anti-LAMP-1 mouse monoclonal antibody (Santa Cruz Biotechnology, clone H5G11) [35] followed by Alexa-488-labeled goat anti-mouse secondary antibody (Molecular Probes). Bacterial nucleoids were then stained with 4′,6-diamidino-2-phenylindole (DAPI). Intracellular bacteria were identified by the absence of extracellular antibody staining and positive staining with DAPI. Colocalization of intracellular bacteria (DAPI^+^, Alexa-594^-^) and LAMP-1 (Alexa-488^+^) was assessed and quantified using a Nikon Eclipse E300 epifluorescence microscope and images capture using a Retiga 2000RV digital camera (QImaging, Surrey, British Columbia) and IPLab software 3.9.4r5 (BD Biosciences, Rockville, MD)

### Electron Microscopy

Bacteria at a multiplicity of infection of approximately 1000∶1 were centrifuged onto HUVEC monolayers at 580 *g* for five minutes. After incubation for the indicated time points, monolayers were washed with PBS, fixed for twenty minutes in Trump's fixative containing 4% formaldehyde and 1% glutaraldehyde, washed in 0.1 M cacodylate buffer, and post fixed in 1% OsO_4_. Monolayers were then washed in maleate buffer, incubated with uranyl acetate, washed again in maleate buffer, dehydrated in ascending alcohol washes, embedded as monolayers in Eponate using an inverted BEEM® capsule (Beem, Inc., Westchester, PA), and sectioned en face. Sections were stained with lead citrate and viewed with the digital image acquisition system on the Jeol (Tokyo, Japan) MEM-1011, transmission electron microscope.
